# New Deployable Expandable Electrodes in the Electroporation Treatment in a Pig Model: A Feasibility and Usability Preliminary Study

**DOI:** 10.3390/cancers12020515

**Published:** 2020-02-23

**Authors:** Francesco Izzo, Franco Ionna, Vincenza Granata, Vittorio Albino, Renato Patrone, Francesco Longo, Agostino Guida, Paolo Delrio, Daniela Rega, Dario Scala, Roberto Pezzuto, Roberta Fusco, Elio Di Bernardo, Valeria D’Alessio, Roberto Grassi, Deyanira Contartese, Raffaele Palaia

**Affiliations:** 1 Division of Surgical Oncology, Hepatobiliary Unit, ISTITUTO NAZIONALE TUMORI–IRCCS-FONDAZIONE G. PASCALE, NAPOLI, ITALIA, Via Mariano Semmola, 80131 Naples, Italy; v.albino@istitutotumori.na.it (V.A.); r.palaia@istitutotumori.na.it (R.P.); 2 Division of Surgical Oncology, Maxillo-Facial Unit, ISTITUTO NAZIONALE TUMORI–IRCCS-FONDAZIONE G. PASCALE, NAPOLI, ITALIA, Via Mariano Semmola, 80131 Naples, Italy; f.ionna@istitutotumori.na.it (F.I.); f.longo@istitutotumori.na.it (F.L.); a.guida@istitutotumori.na.it (A.G.); 3 Division of Radiodiagnostic, ISTITUTO NAZIONALE TUMORI–IRCCS-FONDAZIONE G. PASCALE, NAPOLI, ITALIA, Via Mariano Semmola, 80131 Naples, Italy; v.granata@istitutotumori.na.it; 4 Division of General and Oncologic Surgery, Department of Cardiothoracic Sciences, UNIVERSITA’ DEGLI STUDI DELLA CAMPANIA LUIGI VANVITELLI, NAPOLI, ITALIA, 80131 Naples, Italy; dott.patrone@gmail.com; 5 Division of Surgical Oncology, Colo-Rectal Unit, ISTITUTO NAZIONALE TUMORI–IRCCS-FONDAZIONE G. PASCALE, NAPOLI, ITALIA, Via Mariano Semmola, 80131 Naples, Italy; p.delrio@istitutotumori.na.it (P.D.); d.rega@istitutotumori.na.it (D.R.); d.scala@istitutotumori.na.it (D.S.); robertopezzuto@gmail.com (R.P.); 6 Research & Development Division, Igea SpA, Via Casarea 65, Casalnuovo di Napoli, 80013 Naples, Italy; r.fusco@igeamedical.com (R.F.); e.dibernardo@igeamedical.com (E.D.B.); v.dalessio@igeamedical.com (V.D.); 7 Division of Radiodiagnostic, UNIVERSITA’ DEGLI STUDI DELLA CAMPANIA LUIGI VANVITELLI, NAPOLI, ITALIA, Via Miraglia, 80143 Naples, Italy; roberto.grassi@unicampania.it; 8 Laboratory Preclinical and Surgical Studies, IRCCS–ISTITUTO ORTOPEDICO RIZZOLI, Via di Barbiano 1/10, 40136 Bologna, Italy; deyaniracontartese@libero.it

**Keywords:** electroporation, liver, oral cavity, colon-rectal district, new electrodes

## Abstract

The aim of the study is to evaluate the usability aspects of new deployable, expandable, electrode prototypes, in terms of suitability solutions for laparoscopic applications on the liver, endoscopic trans-oral and trans-anal procedures, electroporation segmentation in several steps, mechanical functionality (flexibility, penetrability), visibility of the electrode under instrumental guidance, compatibility of the electrode with laparoscopic/endoscopic accesses, surgical instruments, and procedural room and safety compatibility. The electroporation was performed on an animal model (Sus Scrofa Large White 60 kg) both in laparoscopy and endoscopy, under ultrasound guidance, and in open surgery. Electrodes without divergence, with needles coming out straight, parallel to each other, and electrodes with peripheral needles (four needles), diverging from the electrode shaft axis (electrode with non-zero divergence) have been tested. To cause an evaluable necrosis effect, the number of electrical pulses was increased to induce immediate liver cell death. Histological samples were analyzed by staining with Haematoxylin/Eosin or by immunohistochemical staining to confirm complete necrosis. The prototypes of expandable electrodes, tested in laparoscopy and endoscopy and in open surgery, respectively, are suitable in terms of usability, electroporation segmentation in several steps, mechanical functionality (flexibility, penetrability), visibility under instrumental guidance, compatibility with laparoscopic/endoscopic accesses, surgical instruments and procedural room safety, patient safety (no bleeding and/or perforation), and treatment efficacy (adequate ablated volume). Electroporation treatment using new deployable expandable electrode prototypes is safe and feasible. Moreover, electrode configurations allow for a gradual increase in the ablated area in consecutive steps, as confirmed by histology and immunohistochemistry.

## 1. Introduction

The clinical use of electroporation (EP) techniques has spread in the last 10 years in Europe and the USA. The EP technique, used alone as Irreversible Electroporation (IRE), or in association with anticancer drugs (electrochemotherapy, ECT), is effective in the treatment of primary and metastatic tumors [1,2,3,4]. 

The EP treatment is not associated with a change in the temperature of the exposed tissue, except for near the needles of the electrodes [5,6]; the treatment can be performed in “noble” structures, such as vessels and nerves, without risk of complication. Moreover, the collagen and vascular tissue structure do not undergo coagulative degeneration as observed with hyperthermic procedures, such as radiofrequency (RFA), microwave (MWA), high-intensity focused ultrasound (HIFU), and laser therapy [7]. RFA and MWA are the two most commonly used techniques for solid organs treatment [7,8]. They have the advantages of greater volume of cellular necrosis and lesser susceptibility to heat dissipation effects due to the presence of neighboring vessels. The main disadvantage is the increased risk of damaging nearby non-target organs [7,8]. However, for irreversible electroporation, it was reported that the increase of pulse application could determine both an efficacy increase of the treatment and associated thermal damage [5,6], limited to the area around the electrodes [5,6,9]. This effect is minimized in cases of reversible electroporation, considering an electric protocol, according to European Standard Operating Procedures of Electrochemotherapy (ESOPE) [10,11,12], which consists of 8 pulses of 100 µs, divided into two groups of four, with inversion of the applied voltage, and an electric field of 1000 V/cm. 

The exact positioning of the electrodes is essential to prevent damage to the surrounding healthy tissue, critical vascular structures, and/or adjacent organs. The use of EP is possible and effective in any tissues; just consider that it is currently used mainly to the skin and to the subcutaneous tissue, where it has filled a “therapeutic vacuum” [10,11]. The efficacy of ECT and IRE has already been demonstrated in metastasis of non-superficial tumors, such as liver cancer [13,14,15,16,17,18,19], rectal cancer [20,21,22], primitive pancreatic cancer [23,24,25,26,27,28], esophageal tumors [28], and bone metastasis [29].

Despite these undoubted advantages, the clinical use and the operative technique for the treatment of tumor lesions, located in areas that are not superficial or immediately reachable, can be complex and prolonged unless the laparotomy is used. Percutaneous EP is not always possible, and the use of EP with minimally invasive techniques or endoscopic is not yet available. A multipolar probe with telescopic needle electrodes for intracorporal IRE and percutaneous ECT treatments already exists for minimally invasive treatment of malignant liver tumors [30]. Intraluminal catheter-directed IRE is feasible and safe for full-thickness ablation of the normal porcine common bile duct. [31]. EP has been already used with an endobronchial catheter-based to treat peribronchial tumors, in combination with cisplatin, showing the feasibility and safety of this approach [32]. A new expandable electrode has already been used to treat experimental rat brain tumors, showing a high efficacy and absence of associated toxicity and morbidity [33]. The optimization of a novel electrode device developed for electrotransfer of antineoplastic drugs and genes to intracranial tumors in humans has led to both clinical performance and geometrical tolerance improvement [34,35]. A phase I clinical study on brain tumors confirmed ECT to be an effective treatment method with limited toxic effects on normal tissue [36]. At Cork Cancer Centre (University of Cork, Cork, Ireland), the first endoluminal electrodes have been developed, with the aim to treat colorectal, gastric, and esophageal tumors [15], and its effectiveness and safety have been demonstrated on a spontaneous canine colorectal cancer study [36]. A phase I/II clinical trial in non-surgical patients with colorectal cancer is currently ongoing (NCT03040180). The technological limitations of EP are linked to the basic principles on which the EP of a tissue is based. The tissue to be submitted to reversible EP for electrochemotherapy (using the standard electric protocol with 8 pulses of 100 µs) should be covered homogeneously by the electric field throughout its volume [37]. Therefore, the active conductive part of the electrodes should be inserted at least to the depth of lesion (or deeper) and the electrodes must be parallel between them to homogeneously cover the surface/volume of the tissue. These prerequisites are not easily fulfilled when tumor nodules to be treated are not easily accessible: hollow or visceral organs. These problems have been solved in the classic ablation techniques (for example, radiofrequency) using an electrode container that, once inserted into the tumor nodule, can dislocate or expand single electrodes that are inflicted in the tissue, and cause degenerative coagulation through heat. This approach is not feasible in the case of EP; in fact, if the electrodes are displaced and expanded, as in the case of radiofrequency, the parallelism is lost and there is no guarantee of obtaining the EP of all cells of the tumor nodule. Furthermore, the electric field applied to the conductive part, due to the divergence between the needle couples, would be extremely inhomogeneous, too high in the nearest part of the electrodes, with the risk of short circuit, and too low in the most distal part, leaving some areas of the tumor not properly treated. However, to completely exclude that electrodes without divergence determine an inhomogeneous electric field, is not possible. 

The identified solution provides that the conductive part of the needles contained in the electrode vary according to the divergence of the needles, and that the EP of the tumor nodule occurs through successive steps, realizing a treatment segmentation. This solution allows the use of a single electrode of small size that contains the needles. The problem of the inhomogeneity of the electric field is overcome by limiting the conductive part of the needle; in this way, the variability of the electric field along the conductive part is reduced.

The electrodes have been tested on an animal model, with the aim to evaluate the feasibility and usability of the EP treatment performed with these new electrodes in different districts: liver with both laparoscopic and open approach, oral cavity, and colorectal lumen using, respectively, the trans-oral and trans-anal pathway with an endoscopic approach. The main usability aspects of electrode prototypes included: 1. suitability solution for specific application and of EP segmentation in several deployments; 2. mechanical functionality (flexibility, penetrability); 3. visibility of the electrode under radiological guidance; 4. compatibility of the electrode with surgical accesses and instruments; 5. procedural room compatibility and safety.

## 2. Materials and Methods

### 2.1. Animal Model

This study was conducted following all applicable international, national, and/or institutional guidelines. The in vivo experiments have been conducted respecting the bio-ethic principles and the current Italian (art. 31 D.L. 26/2014) and European regulations. The use of the animal model was authorized by the Ministry of Health (n. 15/2019-PR), within the Research Project “Expandable electrode for electroporation of tissues”.

The animal (one specimen of Female Sus Scrofa Large White, 60 kg of weight) was anesthetized according to the following anesthetic/analgesic protocol: zoletil anesthesia 50/50 0.5 mL/kg intramuscular (i.m.) + propofol 6 mg/kg intravenous (i.v.) + ketamine 10 mg/kg i.v. + sevorane 2% by inhalation; butorphanol analgesia 0.1–0.4 mg/kg i.m. or i.v. 

### 2.2. Electrodes Description

**Figure 1:**
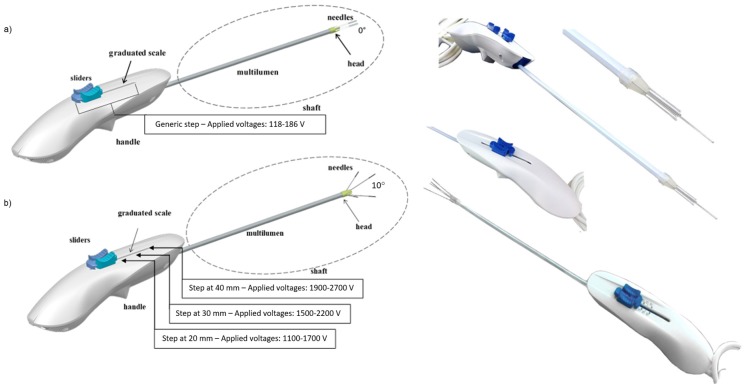
Rendering representation and real realization of deployable expandable electrodes with (**a**) zero and (**b**) non-zero divergence (10° of divergence between peripheral needles and electrode shaft axis). In the deployable expandable electrode without divergence, the applied voltage is 118 V between each peripheral needle, and the central one, and 186 V between two consecutive peripheral needles. In the deployable expandable electrode with 10° of divergence, the applied voltage depends on the needle deployment: at 20 mm the applied voltages are 1100 V and 1700 V (for side and diagonal d of the electrode, respectively); at 30 mm, the applied voltages are 1500 V and 2200 V; at 40 mm the applied voltages are 1900 V and 2700 V. The insulation layer of the electrodes is fixed. The voltages are defined in order to obtain an electric field ≥1500 V/cm for irreversible electroporation.

The EP was made using deployable expandable electrodes (Figure 1).

The electrode consists of: a shapeable shaft made up of five needles (one central needle and four peripheral needles) in medical steel, which run in a multi-lumen, and whose divergence is determined by a head, a handle that allows the gripping of the device, and in which the needles movement system is implemented, and a cable and a connector for the electrical connection to the electroporator.

There are different models of electrodes that differ in the length of the shaft, the divergence of the peripheral needles related to the electrode shaft axis, and for the maximum exposure of the needles.

The configuration with five needles has been chosen to reduce the applied voltages and allow implementing several variants with different divergences between peripheral needles and the electrode shaft axis. The needles constitute the active part of the electrode and, inserted in the target lesion, allow the delivering of the electrical impulses into the target area. The needles are adjustable in their insertion depth by means of a handling system implemented in the handle. The needles have an insulating polymeric sheath, which limits the conductive part exposed (active part of the electrode) at 2 cm (see Table 1). By means of a graduated scale present on the handle itself, it is possible to adjust the insertion depth of the needles up to 4 cm. At different steps, the distances between each needle depend on the initial distance between each needle, which is determined by the design of the shaft, and the divergence between the peripheral needles and the electrode shaft axis. Particularly, the distance between each peripheral needles and the central one, which represents the semi-diagonal of the electrode (d/2), the distance between two opposite peripheral needles, which represents the diagonal of the electrode (d), and the distance between two consecutive peripheral needles, which represents the side of the electrode (side), are respectively calculated by solving the following formulas:

(1) $$d/2=\left(e+h_{t}\right)\tan\alpha +\frac{i}{2}-\emptyset_{\mathrm{needle}},$$

(2) d=2·d/2,

(3) $$side=\sqrt{2}\cdot \left(d/2\right)-\left(1-\sqrt{2}\right)\cdot \emptyset_{needle},$$

where: the parameters *h_t_* and *i* are fixed by the design of the shaft and measure, respectively, 3 mm and 3.26 mm; e is the exposition of the needles at each step; α is the divergence angle of the specific electrode variant; Ø_needle_ is the diameter of needles, which must be subtracted, in order to define the correct voltage to be applied on the inner space among each needles couple, and measure 0.45 mm.

**Figure 2:**
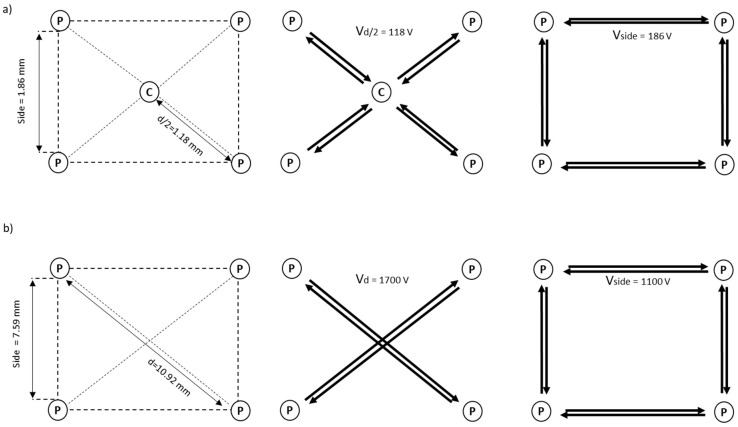
Examples of geometrical configurations of (**a**) an expandable electrode without divergence with four peripheral needles (P), and one central needle (C), and (**b**) an expandable electrode with 10 degrees of divergence with four peripheral needles (P) and without the central one. In the configuration (a) the side and semi-diagonal sizes and the applied voltages for each side (V_side_ = applied voltage between two consecutive peripheral needles) and for each semi-diagonal (V_d/2_ = applied voltage between each peripheral needle and the central one) are shown. In the configuration (b) the side and diagonal sizes (d), and the applied voltages for each side, and for each diagonal (V_d_ = applied voltage between two opposite peripheral needles), in the case of 20 mm of needles deployment, are shown.

Consequently, the electric parameters at each step are different, based on the electrode divergence (see Figure 1). For example, in the deployable expandable electrode without divergence (*α* = 0°, see Figure 2), the side of the electrode measures 1.86 mm, while semi-diagonal measures 1.18 mm; therefore, the applied voltages are 118 V at the semi-diagonal of the electrode and 186 V at the side of the electrode.

In the deployable expandable electrode with 10° of divergence and without the central needle (see Figure 2), the applied voltage depends by the needles deployment: at 20 mm the applied voltages are 1100 V and 1700 V at the side and the diagonal of the electrode respectively (side = 7.59 mm, d = 10.92 mm); at 30 mm the applied voltages are 1500 V and 2200 V (side = 10.08 mm, d = 14.44 mm); at 40 mm the applied voltages are 1900 V and 2700 V (side = 12.58 mm, d = 17.98 mm). In these experiments, the voltages of divergent electrode prototypes are defined in order to obtain an electric field ≥1500 V/cm for irreversible electroporation.

The central needle and the peripheral needles can be moved independently by using the dedicated cursors, in order to fix the target lesion with the central needle. The possible scenarios are:

- Insertion: first move the central needle and then advance the peripheral needles or, alternatively, move the cursor of the peripheral needles to move the entire set of needles;

- Extraction: first move the peripheral needles and then the central needle or, alternatively, move the entire set of needles back by moving the central needle cursor. 

**Figure 3:**
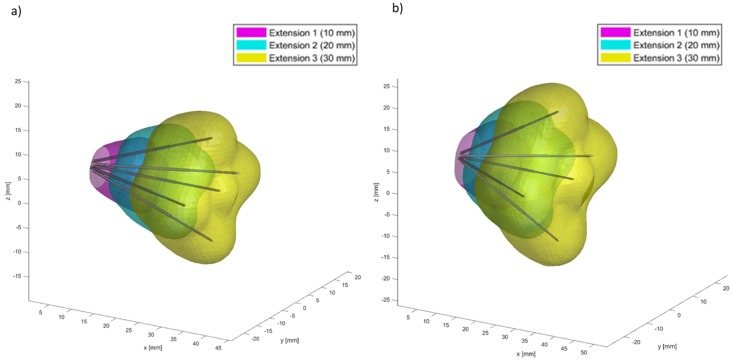
Finite Element Method (FEM) simulations for a deployable expandable electrode with a divergence angle of (**a**) 20° and of (**b**) 30° considering three electroporation steps (10 mm, 20 mm, and 30 mm).

Finite Element Method (FEM) simulations using Comsol Multiphysics (v5.2, Stockholm, Sweden), controlled from Matlab (MathWorks, Natick, MA, USA) by means the LiveLink interface, were carried out to verify that the electric field distribution was 1000 V/cm between the tips of needles. The minimum electric field considered in the simulations for a successful electroporation was 400 V/cm; the tissue was considered as homogeneous with a conductivity range of 0.1–0.3 S/m; the tissue conductivity was considered as isotropic and increased as a function of the maximum magnitude of the electric field in each point of the space [38,39,40]. The volumetric coverage, in multiple deployments, using two configurations with different divergence and with five needles is shown in Figure 3. The figure reports FEM simulations for a deployable expandable electrode with a divergence angle of (a) 20 degrees and (b) 30 degrees, considering three electroporation deployments (10 mm, 20 mm, and 30 mm).

### 2.3. Operative Electroporation Procedure

To evaluate the feasibility and usability of the proposed electrodes, the complete operative electroporation procedure has been performed on one animal using three electrodes configuration for each of the following districts: liver with both laparoscopic and open approach, endoscopic using the trans-oral and trans-anal pathway. By using a specific usability questionnaire, three users for each electrode configuration used and for each district treated have reported their opinions concerning the different operative procedure aspects and the electrode characteristics.

To assess the treatment efficacy of the proposed electrodes, irreversible electroporation was executed on liver.

#### 2.3.1. Liver Intervention in Laparoscopy or Laparotomy Approach

For EP applications on liver, 3 electrode prototypes were used: one electrode with zero divergence and two electrode prototypes with different divergence (20° and 10°) and with 5 and 4 needles, respectively (see Table 1). The configuration with 4 electrodes did not have the central needle. The treatment was carried out under ultrasound guidance. The irreversible EP treatment was performed, both in laparoscopic procedure and in open surgery, using the Cliniporator Vitae (Igea SpA, Carpi, Italy) and setting the following electrical parameters to obtain irreversible electroporation: 80–120 pulses, as indicated in the Table 1; 100 µs of pulse duration; 5 kHz of pulse frequency; variable voltage based on electrode prototype and on deployment of needles (see Figure 1 and Table 1).

A Computed Tomography (CT) with iodate contrast medium injection (non-ionic, water solution contrast medium—Iopamiro, Bracco, Milan, Italy) was performed 3 h after the treatment. The animal was then sacrificed and liver specimens removed for vital staining (Tetrazolium) and/or histology and immunohistochemistry evaluation. 

#### 2.3.2. Endoscopic Trans-Oral and Trans-Anal Approach

For oral approach, two samples of the electrode with zero divergence and with different shaft rigidity have been used. 

For both trans-oral and trans-anal approaches, a model of electrode with 10° of divergence has been used (see Table 1).

A CT with iodate contrast medium was performed during the procedure of needles insertion and extraction. 

### 2.4. Imaging Data Analysis

The calculation of the volume of ablated areas was performed on the CT imaging data. The numerical evaluation of the volume was conducted using two approaches:

- Calculation of the volume of the smallest ellipsoid containing the ablated area. The ellipsoid is generated by considering, as its axes, the maximum diameters of the area in the 3 orthogonal planes. The volume of the ellipsoid is given by:
  (4) $$V_{ellipsoid}=\frac{4}{3}\pi abc,$$
  where *a*, *b,* and *c* are the semi-axes of the ellipsoid (or the radii of the area in the 3 planes).
- Real calculation of the segmented area volume using the CT imaging parameters: total number of pixels contained in the segmented area (n_pixel_), horizontal pixel spacing (PS_o_), vertical pixel spacing (PS_v_), space between slice (SBS), slice thickness (ST). By considering the presence of a possible gap (SBS - ST) between one slice and the next, the volume is given by:
  (5) $$V_{CT}=n_{pixel}\cdot PS_{o}\cdot PS_{v}\cdot \left[ST+\left(SBS-ST\right)\right]=n_{pixel}\cdot PS_{o}\cdot PS_{v}\cdot SBS,$$

### 2.5. Histological Analysis

Tissues for histopathological analysis were macroscopically and histologically examined. The specimens were obtained sectioning the liver along the orthogonal plane. Each sample contains both the central part of the region and the surrounding area; includes an adjacent rim of non-ablated hepatic parenchyma. The tissues were processed and embedded in paraffin and the sections obtained were stained with Hematoxylin/Eosin (Sigma Aldrich, Milan, Italy) for subsequent histological evaluations. A mean number of 6 slides per tumor was analyzed (range 2 to 12 slides). The presence of necrosis (type and extension), inflammatory infiltrates, changes in blood vessels and biliary ducts, in both the treated and non-treated areas, and eventual residual vital tissue (amount and location) were analyzed. A light microscopy (Olympus BX51) was used to observe the slides, and the Aperio digital scanner (AperioScanscope CS System, Aperio Technologies, Vista, CA, USA) was used to capture representative images from each slide using under different magnifications specified in the figure legends.

### 2.6. Immunohistochemical Analysis

Additional slides for each specimen were immunostained for caspase-3 (CASP3) according to standard protocol. Negative controls, obtained omitting the primary antibody, were included to check proper specificity and performance of the applied reagents. 

## 3. Results

One irreversible EP treatment for each electrode configuration was performed on the liver using both laparoscopic approach and open surgery. The treatment result was evaluated in terms of the presence of necrosis. The ablated volume is reported in Table 2. 

The opinions in terms of feasibility and usability formulated by clinicians are reported in Table 3. Each electrode model, tested in laparoscopic/endoscopic surgical approaches and in open surgery, with zero and non-zero divergence, are suitable in terms of usability, mechanical functionality (flexibility, penetrability), visibility of the electrode under radiological guidance, compatibility of the electrode with specific surgical accesses, patient safety (no bleeding and/or perforation) and treatment efficacy (adequate ablated volume). The ergonomics of the handle is satisfactory and adequate for the treatments carried out. The flexibility and the shape of the invasive element have proven to be suitable for satisfying the required performance. The thrust of the needles does not alter the shape pre-imprinted to the shaft and has not shown particular resistance during the needles insertion into the treated tissues (liver, tongue base, and rectum).

**Figure 4:**
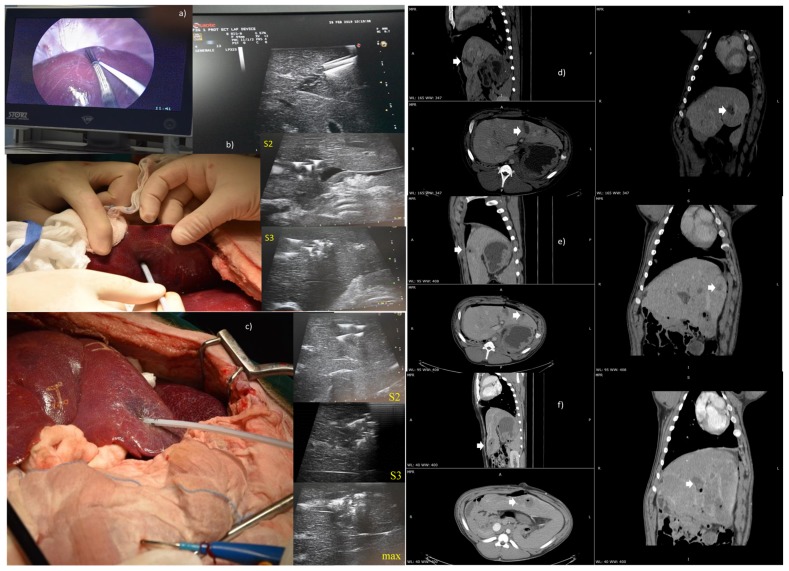
(**a**) Treatment performed by the laparoscopic access and ultrasound images used as a guide for the insertion of a 5-needle expandable electrode with zero divergence; (**b**) treatment in open surgery with a 5-needle expandable electrode prototype and ultrasound vision of the area treated in the two deployments (S2–S3); (**c**) treatment in open surgery using a 4-needle expandable electrode prototype (without central needle) and ultrasound vision of the area treated in the three deployments; (**d**) CT Multiplanar reconstructions of the ablated area with the 5-needles expandable electrode with zero divergence; (**e**) CT Multiplanar reconstructions of the ablated area with the 5-needles expandable electrode prototype (20° of divergence) and (**f**) with the 4-needles expandable electrode prototype (10° of divergence).

Figure 4 reports the treatment performed using a 5-needle expandable electrode prototype, both with the laparoscopic approach (model with zero divergence) and in open surgery (model with 20° of divergence), with ultrasound images, to guide the electrode insertion (two steps of insertion were performed). Moreover, the treatment was performed in open surgery using a 4-needle expandable electrode prototype (4 peripheral needles without central one, 10° of divergence), with ultrasound vision of the area treated in three deployments. In addition, CT multiplanar reconstructions of the ablated area, with both the 5-needle and 4-needle expandable electrode prototypes, are shown.

**Figure 5:**
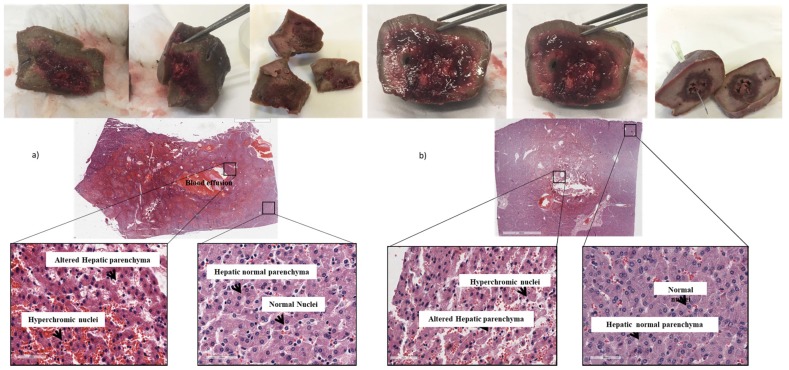
(**a**) Macroscopic images and histological analysis of specimens treated with electrode with zero divergence. (**b**) Macroscopic images and histological analysis of specimens treated with the 4-needle expandable electrode.

**Figure 6:**
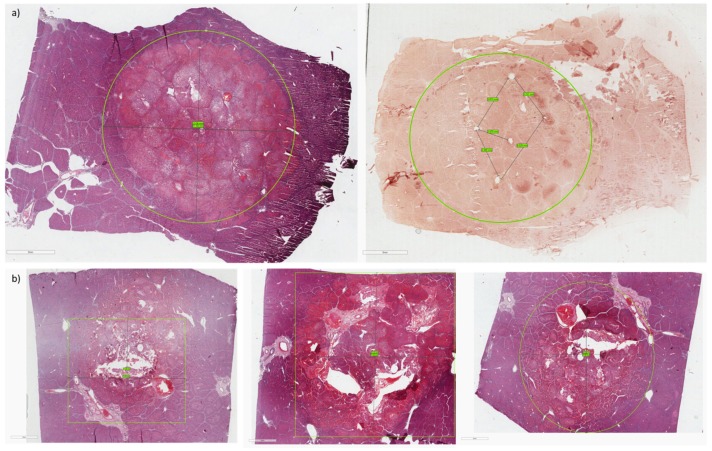
(**a**) Histological and immunohistochemical analysis on specimens treated with electrode with zero divergence; (**b**) Histological and immunohistochemical analysis on specimens treated with the 4-needle expandable electrode. In the circle or square it is possible to observe the size of the necrosis area.

The histological examination also demonstrated the efficacy of the treatment, highlighting a difference between treated (central part) and untreated (surrounding part) hepatic tissue. In particular, in the treated tissue the presence of hypertrophic nuclei, blood effusion, and altered and damaged hepatic parenchyma was observed. In the untreated tissue, the presence of normal nuclei and intact hepatic parenchyma was observed (Figure 5). The immunohistochemical staining also confirmed the presence of necrosis in the treated tissues, with apoptotic cells characterized by a positive antibody-reaction, compared to untreated tissues where the antibody reaction was negative (Figure 6b). The images obtained with both histological and immunohistochemical staining allowed to observe and measure the extension of necrosis area in the central part of tissues (Figure 6).

**Figure 7:**
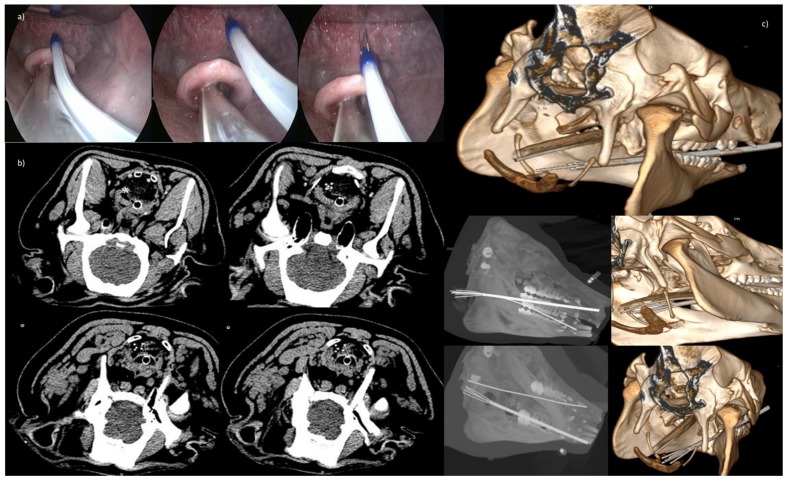
(**a**) Trans-oral application for intraluminal access to upper airways with the 5-needle expandable electrode (20° of divergence); (**b**) CT transversal sections in the 3 deployments (2 cm, 3 cm, 4 cm); (**c**) Volume rendering with central needle exposure of 4 cm and peripheral needles not exposed, and below MIP and Volume rendering, with central needle exposure of 4 cm, and peripheral needle exposure for 3 cm and for 4 cm, respectively.

Figure 7 reports the use of the 5-needle expandable electrode (20° of divergence) in a trans-oral application for intraluminal access to upper airways. CT transversal sections in different needles steps are also shown in the panel with the CT Maximum Intensity Projection (MIP) and Volume rendering.

**Figure 8:**
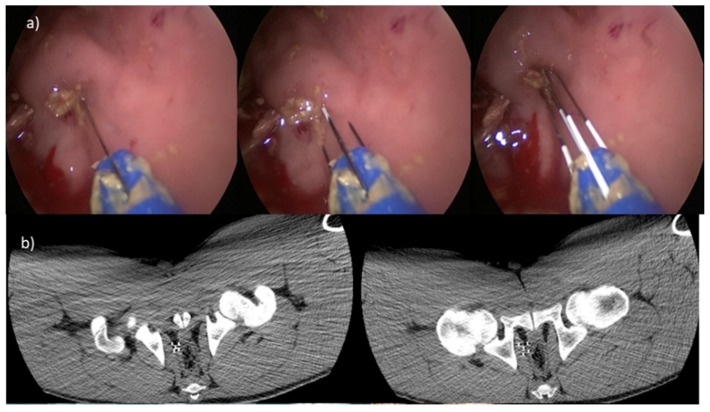

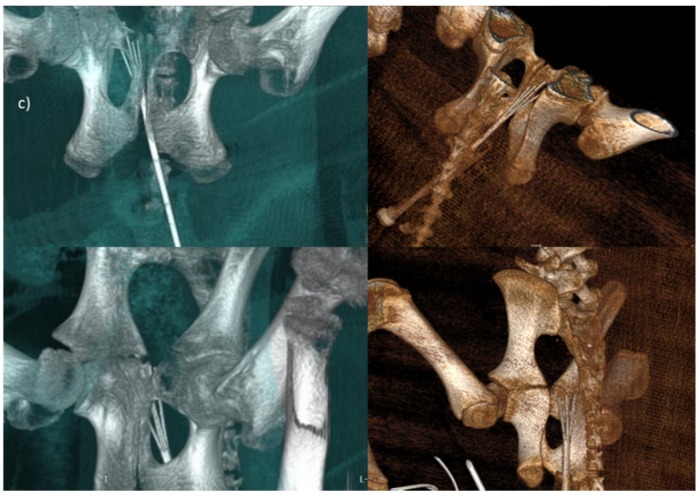
(**a**) Application on rectum with the trans-anal endoscopic procedure and the 5-needle expandable electrode (20° of divergence; (**b**) CT transversal sections in the two deployments: on the left, peripheral needles with exposure of 3 cm; on the right side, peripheral needles exposure of 4 cm; (**c**) volume rendering: in the top, deployment of central needle of 4 cm and peripheral needle exposure of 3 cm; down, central needle and peripheral needles exposure of 4 cm.

Figure 8 reports the application on rectum using the trans-anal endoscopic approach and the 5-needle expandable electrode (20° of divergence). CT transversal sections in different needles steps are also shown in the panel with the CT MIP and Volume rendering.

## 4. Discussion

Treatment with ECT of deep-seated nodules, either percutaneously or during laparoscopic/endoscopic procedures, is at its early stages. An accurate placement of the electrodes, as well as image guidance, improve ECT treatment, successfully minimizing risk of a new intervention. Laparoscopic/endoscopic ECT of solid organs is a novel, minimally invasive treatment modality, and potentially very effective [41]. Laparoscopic surgery has some advantages versus open surgery, such as faster recovery, reduced hospital stay, and lower hospital costs. Many studies show that laparoscopic cancer surgery is associated with better outcomes in terms of reduced surgical complications and perioperative morbidity [42,43,44,45].

ECT treatment is generally well accepted by patients because it improves quality of life, in terms of pain and bleeding reduction. Need for medical/paramedical care has also been decreased with consequent cost reduction [46]. 

The first endoluminal electrodes have been developed at Cork Cancer Centre at the University of Cork, Ireland. A preclinical study on spontaneous canine colorectal cancer showed that EndoVe device is safe and effective in tumor resolution [36]. Recently, results of the first human clinical trial of phase I on endoscopic ECT for advanced esophageal cancer have been reported [28]. No serious complications were observed and, in five out of six treated patients, tumor response was confirmed by gastroscopy. In two cases, reduction of total tumor mass was confirmed with 18- fluorodeoxyglucose (FDG) positron emission tomography (PET)/Magnetic Resonance Imaging (MRI) [28]. Therefore, an endoscopic treatment for colorectal cancer has been investigated and the preliminary results confirmed that the entire procedure is minimally invasive and can be done on an outpatient basis [21]. 

The main impact indicators for products developed in the project are constituted by the high efficacy of ECT in the local treatment of tumors, its limited toxicity, and its high acceptability by the patients. The technology presented here will allow the use of EP in minimally invasive laparoscopic and endoscopic procedures, greatly expanding the possibilities of use the EP technique. It will be possible to offer the therapy to a greater number of patients, e.g., those patients for whom the surgery is currently not possible, as well as for those who are not candidates for thermal ablation.

The tested prototypes were suitable for laparoscopic/endoscopic treatments, and for the use in combination with laparoscopic ports, and for endoluminal optical instrumentation. This will allow huge savings on healthcare costs in terms of medical and paramedical personnel, as well as a marked improvement of the clinical conditions of the treated subjects [46]. The miniaturization of the electrode, the divergence of needle, and the segmental treatment allow the use of EP in endoscopic approach for treating anatomical areas excluded until now, such as the oral cavity and the upper respiratory tract, the esophagus of the digestive tract, and the colon-rectum. The risk of accidental blood vessel perforation and bleeding, reported in clinical practice for the use of tined needles, is minimized using the proposed electrodes, due of the EP effect. In fact, EP induces vascular blockade avoid bleeding [47], does not expose to the risk of perforation since the collagen structure of the tissue remains intact after insertion of needles of size <0.7 mm, and does not increase the risk of infection of the abdominal wall or cavity.

Laparoscopic electrodes can be used for not resectable liver metastasis, pancreatic tumors, and locally advanced renal carcinomas. Only 25% of patients with colorectal liver metastases (112,000 patients in Europe each year) are eligible for surgery. The rest of the patients (84,000) are managed with palliative therapies (RF, Microwave, chemoembolization, hepatic perfusion) [48]; 10% of these patients could be treated with ECT (8400). The European incidence of locally advanced pancreatic cancer is of 104,000 new cases per year, and most of them (80%) are not suitable for surgery; for these patients, the survival rate is only 1% at five years [49]. With the new electrode, it will be possible to treat at least 10% to 20% of the entire number of patients.

Wandel et al. [50] reported the use of a single insertion bipolar needle for liver IRE application in a pig model. They concluded that bipolar IRE ablation zones can be increased with repetitive high voltage and greater pulse widths, accompanied by either judicious instillation of hypotonic fluids or internal electrode perfusion to minimize unwanted electric arcing. However, the electrode proposed in our study should be, based on our knowledge, the first expandable and divergent model that allows ECT applications using a segmental electroporation approach.

## 5. Conclusions

In conclusion, the tested prototypes are suitable for laparoscopic/endoscopic treatments and for the use in combination with laparoscopic ports and endoluminal optical instrumentation. Moreover, the electrode configurations and the segmental electroporation allow for a gradual increase in the ablated area in consecutive steps.

## Figures and Tables

**Table 1:** Characteristics of tested electrodes with 5 and 4 needles, including technical proprieties (shaft diameter Ø, shaft length, divergence, maximum deployment and active part), geometry configuration and electric parameters.

Electrode Model		Geometry Configuration		Electric Parameters	
**Expandable electrode prototype with 5 needles with zero divergence**					
Ø shaft [mm]	5	Side [mm]	1.86	V_side_ [V]	186
Shaft Length [cm]	50	Semi-diagonal d/2 [mm]	1.18	V_d/2_ [V]	118
Divergence [°]	0			Pulses Number	80
Maximum Deployment [cm]	4				
Active Part [cm]	2				
**Expandable electrode prototype with 5 needles with non-zero divergence**					
Ø shaft [mm]	5	Deployments	S2, S3 *	V_side_ (S2) [V]	1200
Shaft Length [cm]	20	Side–d/2 (S2) [mm]	13.69–9.55	V_d/2_ (S2) [V]	900
Divergence [°]	20	Side–d/2 (S3) [mm]	18.84–13.19	V_side_ (S3)[V]	1700
Deployment [cm]	4			V_d/2_ (S3) [V]	1100
Active Part [cm]	2			Pulses Number	120
**Expandable electrode prototype with 4 needles with non-zero divergence**					
Ø shaft [mm]	5	Deployments	S2, S3, max *	V_side_ (S2) [V]	1100
Shaft Length [cm]	20	Side–d (S2) [mm]	7.59–10.92	V_d_ (S2) [V]	1700
Divergence [°]	10	Side–d (S3) [mm]	10.08–14.44	V_side_ (S3)[V]	1500
Deployment [cm]	4	Side–d (max) [mm]	12.58–17.98	V_d_ (S3) [V]	2200
Active Part [cm]	2			V_side_ (max) [V]	1900
				V_d_ (max) [V]	2700
				Pulses Number	80

* S2 = second step corresponding to a 20 mm deployment; S3 = third step corresponding to a 30 mm deployment; max = fourth step corresponding to a 40 mm deployment; Side = distance between two consecutive peripheral needles; d = diagonal (that is the distance between two opposite peripheral needle in the electrode without central needle); d/2 = semi-diagonal (that is the distance between each peripheral needle and the central needle in the electrode with central needle); V_side_ = Applied voltage between two consecutive peripheral needles; V_d_ = Applied voltage between two opposite peripheral needles; V_d/2_ = Applied voltage between each peripheral needles and the central needle; The insulation layer for each variant of electrode is fixed. In these experiments, the voltages of divergent electrode prototypes are defined in order to obtain an electric field ≥ 1500 V/cm.

**Table 2:** Volume of ablated areas.

	2a [cm]	2b [cm]	2c [cm]	V_ellipsoid_ [cm^3^]	n_pixel_	V_CT_ [cm^3^]
Expandable electrode prototype with 5 needles and zero divergence	2.05	1.53	1.77	2.91	990	2.38
Expandable electrode prototype with 4 needles and a 10° divergence	2.6	2.14	2.14	6.23	2911	6.99
Expandable electrode prototype with 5 needles and a 20° divergence	3.04	2.49	2.57	10.19	4218	10.13

a,b,c = semi-axes of the ellipsoid (or the radii of the area in the 3 planes). The semi-axes were calculated considering the three maximum diameters obtained by the Computed Tomography (CT) images in the 3 orthogonal planes; V_ellipsoid_ = volume of the ellipsoid; n_pixel_ = number of pixels contained in the segmented area; V_CT_ = volume calculated using the CT imaging parameters (PS_o_ = 0.9766 mm; PS_v_ = 0.9766 mm; ST = 2.5 mm; SBS = 2.5 mm).

**Table 3:** Clinicians’ opinions about the feasibility and the usability of the tested electrodes.

	Liver District	Oral Cavity	Anal Cavity
**Usability**	Suitable	Suitable	Suitable
**Appropriateness of the handle**	Suggested a greater tactile feedback to better monitor the needle advancement even by counting the deployments.	Suggested modification of the positioning of the graduated scale relative to the movement of central needle cursor (left cursor). It would be preferable to have the zero position at the upper end of the cursor (i.e., the entire blue element). Suggested a greater tactile feedback to better monitor the needle advancement even by counting the deployments.	Proper handle. It is possible to exploit the shape of the lower half-shell to prepare the electrode (post-insertion) and maneuver it with greater precision.
**Adequacy of the handling system (cursors)**	Excessive sliding of central needle cursor. Suggested greater friction, in order to increase the precision during the movement of the central needle.	Excessive sliding of central needle cursor. Suggested greater friction, in order to increase the precision during the movement of the central needle.	Excessive sliding of central needle cursor. Suggested greater friction, in order to increase the precision during the movement of the central needle.
**Flexibility and shape of the shaft**	Adapted.	Adapted to the anatomy of the oral cavity. The not excessive flexibility of the cannula allows a better holding of the shape given to the shaft.	Adapted to the anatomy of anal cavity.
**Needles penetration**	No critical issues emerged.	No critical issues emerged.	No critical issues emerged.
**Electrode compatibility with laparoscopic trocar**	The electrode is compatible with the 5 mm laparoscopic trocar. Not recommended the use of the electrode with laparoscopic trocar with larger diameter. The shaft would have excessive freedom of movement, thus reducing precision during use.	Not evaluated.	The electrode is compatible with the 5 mm single-port trocar. Not recommended the use of the electrode with trocar with larger diameter. The shaft would have excessive freedom of movement, thus reducing precision during use.
**Electrode compatibility with clamps**	The laparoscopic forceps used showed that it is possible to grasp the shaft and move it with adequate stability. Compatible with surgical instruments to imprint an angle to the shaft.	Compatible with surgical instruments to imprint an angle to the shaft.	The laparoscopic forceps used showed that it is possible to grasp the shaft and move it with adequate stability. To shape the shaft using pliers it is necessary that these have a curved shape, to prepare the electrode along the orthogonal direction.
**Criticalities found**	None.	None.	None.
